# Editorial: Rapid detection of fungi, microbial, and viral pathogens based on emerging biosensing technology

**DOI:** 10.3389/fbioe.2022.1067322

**Published:** 2022-11-17

**Authors:** Han-Sheng Chuang, Yu-Jui Fan, Tzong-Rong Ger, Nan-Fu Chiu, Stuart J. Williams, Haim H. Bau

**Affiliations:** ^1^ Department of Biomedical Engineering, National Cheng Kung University, Tainan, Taiwan; ^2^ Medical Device Innovation Center, National Cheng Kung University, Tainan, Taiwan; ^3^ School of Biomedical Engineering, Taipei Medical University, Taipei, Taiwan; ^4^ Department of Biomedical Engineering, Chung Yuan Christian University, Taoyuan, Taiwan; ^5^ Laboratory of Nano-Photonics and Biosensors, Institute of Electro-Optical Engineering, National Taiwan Normal University, Taipei, Taiwan; ^6^ Department of Mechanical Engineering, University of Louisville, Louisville, KY, United States; ^7^ Department of Mechanical Engineering and Applied Mechanics, School of Engineering and Applied Science, University of Pennsylvania, Philadelphia, PA, United States

**Keywords:** biosensing technology, pathogen, fungi, bacteria, viruses

Fungi, microbial, and viral pathogens have been constantly posing serious threats to human society over the past centuries. Notorious examples in history that claimed millions of innocent lives, like the Plague of Justinian in 541 AD, the Black Death in 1347, the Italian Plague in 1629–1631, the Great Plague of London during the 16th and 17th centuries, the Spanish Flu in 1918, and recent COVID-19 pandemic that started in 2019, all resulted from either bacterium or virus outbreaks. However, the adverse situation had never been overturned until the second industrial resolution in the late 19th century and early 20th century that brought up immense science advances. The rapidly progressive technology, for the first, imparted humans some powerful weapons to win the unconventional war on the invisible battlefields. The comprehensive understanding of microbiology renders the science behind the microbes and leads researchers to decipher more traits about their weakness and strength. In the 1990s, the emerging microfabrication became the first cornerstone to bring the lab-on-a-chip style biosensing technology from theory to reality. The central concept is to seek early medical treatments by early diagnosis. Recently, numerous research efforts have been made in mechanical, optical, electrical, biochemical aspects, making the sensing technology more accurate, sensitive, specific, compact, cost-effective, and rapid than their past counterparts. Electrochemistry has been long adopted as a label-free means in most biochemical detections (CesewskiB and Johnson, 2020). Their simple integration with semiconductors promotes their high acceptance in variously electrical platforms. Surface enhanced Raman scattering (SERS) and surface plasmon resonance (SPR) are two advanced optical approaches that can easily achieve highly sensitive detection for trace pathogens (Kurochkin et al., 2020; Park et al., 2022a). However, their dependence on sophisticated analyzing instruments forms a barrier to layman users. Unlike most techniques, cellulose/paper-based lateral flow assays (LFA) need least external support, hence are cost-effective and can be carried out in resource-limited areas (Sohrabi et al., 2022). Unfortunately, the simplicity also limits the accuracy and sensitivity of the LFA on the other way. Notably, a novel biosensing technique taking advantage of the field effect transistors (FET) from the semiconductor industry, termed BioFET, has gradually drawn people’s attention and emerged its potential in the demanding biosensing market. With the continual advancement of the current semiconductor technology, BioFET is believed to achieve unprecedented breakthroughs in all aspects in the future (Panahi et al., 2021). Among the techniques, many of them have been used to assist the detection of pathogens, yet, there still exist challenges that need to be addressed. Considering superbugs and variant viruses become more severe and hard to deal with nowadays, a majority of diagnostic approaches that rely on costly, labor-intensive, bulky, and expert-dependent facilities reflects the facts of low-efficiency, lack of flexibility, and inconvenience in our current medical systems. In this regard, decentralization of diagnosis (i.e., point-of-care testing, POCT) provides a promising solution to this deficiency. With POCT, people can manage their health in a more rapid, cost-effective, and user-friendly way.

After being raged by the COVID-19 pandemic for nearly 3 years, the world has realized the essence of rapid POCT tools in defending the health of our society. In response to the urgent needs and beyond, we edited this Research Topic to disclose the latest developments of various emerging biosensing technologies for rapid detection of pathogenic microbes. This Research Topic collected eight regular research articles and one review article, which cover broad focuses of microbes across *Candida auris*, *Staphylococcus aureus*, Hepatitis B virus, and more. Their research highlights are briefly summarized below to serve as an initial orientation for readers.

Among the accepted papers, five of them are associated with emerging bacteria-related diagnostic approaches. Chen et al., utilized cycling enzymatic amplification based on HRP and TMB to achieve rapid electrochemical microbial quantification and antimicrobial susceptibility (AST) profiling. Bacterial strains, including *Enterobacter cloacae*, *Escherichia coli*, *Klebsiella aerogenes*, and *Klebsiella pneumoniae*, were conducted in this study. Their findings concluded that 6 CFU/ml was the limit of detection (LoD) out of the 500-μl whole blood samples for their proposed method. For AST, they utilized dual-dilution kinetic curves and automatable molecular quantification of species-specific 16S rRNA through the use of an electrochemical sensor to assess microbiological responses to antibiotic exposure. In the final blinded clinical test, four urine samples were investigated at a concentration of 10^5^ CFU/ml. All four results showed good agreements with the CLSI breakpoints. Eventually, they claimed the approach was able to identify all blood borne pathogens in 200–500 μl blood volume in less than 8 h while the total assay time for the uropathogen ID can be as short as 2 h due to high abundance in nature (>10^8^ CFU/ml).

In another study, Zheng et al., reported a deliberate combination of silver nanoparticle (AgNP)-invertase complexes and the personal glucose meter (PGM) to detect the presence of bacteria. The principle is simply based on the competitive binding between PEI-AuNPs and other negatively charged species, which are invertase and bacteria in this case. The increased glucose concentration can be eventually measured by the PGM. By optimizing the operating conditions, they achieved a LoD of 7.59 × 10^2^ CFU/ml. With the same approach, *E. coli* and *S. aureus* both exhibited a good linear relationship between the PGM signal and the bacterial concentration from 10^2^ to 10^7^ CFU/ml. For AST, *E. coli* was treated with four antibiotics. All of them showed good inhibited results after 5-h incubation when the PGM signal was below 27.5 mg/dl. Despite the smart combination of AgNP-invertase complexes and the PGM, the approach appears not to be selective to bacterial strains.

In addition to bacteria, fungi-associated diseases are also commonly occurred in the human society and sometimes can be life-threatening if not been diagnosed in a timely fashion. To address this challenge, Lee et al., proposed a portable droplet magnetofluidic (DM) device along with a DM assay cartridge, termed POC.auris, to carry out the rapid duplex detection of *Candida auris* based on qPCR. Three major *C. auris* clade isolates that originate from South Asia, Africa, and South America were measured with the POC.auris. The measurement result was obtained within 30 min and a LoD of 300 CFU/ml was achieved. Despite the successful demonstration, the authors aimed to keep working on several improvements of the current system to make it a step closer to a clinic-ready product. The anticipated improvements are replacing the mock samples with real clinical ones, replacing the manual off-cartridge cell lysis and cartridge preparation with a simpler workflow, enhancing the sensitivity and turnaround time, adapting to different PCR assays, and reducing the operating cost.

Sharing similar interests in fungal infections with the previous group, Barbosa et al., developed a microfluidic chip combined with fluorescence *in situ* hybridization (FISH) to better monitor the urinary tract infection (UTI), caused by opportunistic pathogens such as *Candida albicans* or non-albicans *Candida species* (NACS). Instead of laborious, time- and reagent-consuming UTI diagnosis, the authors fabricated a microchannel to trap and separate target cells from suspension. A specific peptide nucleic acid (PNA) probe was used to identify the trapped cells and generate fluorescent signal. They believed that their proposed diagnostic method can be a solution herein and adapted to point-of-care detection. The final result showed that *C. tropicalis* detection (∼10^5^ cell/ml) in artificial urine was achieved in 6 h, which is faster than current urine culture method that takes 18–48 h. It should be noted that autofluorescence arising from biological matrices and inorganic debris may form a main challenge in FISH according to the authors’ account.

In the collection of bacterial detection, a reviewer article authored by Chen et al., focuses on the up-to-date developments of aptamer-based biosensors for rapid and sensitive detection of *Staphylococcus aureus*. The authors emphasized timely, rapid and accurate detection of *S. aureus* is of particular significance in this paper. Unlike conventional immunoassays, aptamer is a specific oligonucleotide sequence that features high sensitivity, high specificity, and easy storage. Moreover, aptamers can efficiently and specifically attach to a wide range of targets, including small molecules, ions, peptides, proteins, viruses, bacteria and even cells. Benefit by aptamers, aptasensors have been rapidly emerging in recent years due to their fast, specific, low cost, low sample volume, automated, and portable advantages. To facilitate the introduction, sensors are divided in two categories—optical transduction and electrochemical transduction. The former aptasensors discussed are based on colorimetry, fluorescence, SERS, SPR, and chemiluminescence while the later aptasensors included are based on potentiometry, amperometry/voltammetry, impedance, and conductometry. In the end, some aptasensors in the point-of-care testing applications are discussed. While this review comprehensively explores the recent advances in aptasensors for *S. aureus* detection, some significant challenges regarding aptamers, such as tedious SELEX, biostability, interference effects from metal ions, anions, and antioxidants, reproducibility, and multiplexing detection, still need plenty of efforts to improve in order to achieve future practical use.

Unlike bacteria, viruses develop a different strategy to invade into human body and impair our health. Nucleic acid amplification has always been an effective measure to help clinicians diagnose the viral diseases in the early stage. Unfortunately, the conventional RT-PCR is obvious too tedious to provide handy and timely information. Therefore, Park et al., managed to develop one-step digital RT-PCR for viral RNA detection. Two viruses, tobacco mosaic virus and cucumber mosaic virus, were tested as model viral particles in this study. Instead of a bulky vial used in the conventional RT-PCR, a single unit comprising 20,000 chambers of sub-nanoliter volume was used to achieve multiple amplification reactions. As compared with the conventional RT-PCR, the proposed one-step digital RT-PCR mainly saved the processing time in virus lysis, reverse transcription, and cDNA purification. Finally, their analysis yielded 1,130.2 copies/µl using 10^–2^ μg/nl of viral particles in a 30 min.

Another study conducted by Waller et al., focused on developing a device to address the need for rapid, simple, scalable, and high-throughput multiplex diagnostics in non-laboratory settings. In this paper, the authors demonstrate a multiplex reverse-transcription loop-mediated isothermal amplification (RT-LAMP) coupled with a gold nanoparticle-based lateral flow immunoassay (LFIA) capable of detecting up to three unique SARS-CoV-2 viral gene targets (Orf1ab, Envelope, and Nucleocapsid) in 15 min for RT-LAMP plus 30 min for LFIA. A colorimetric change was used to confirm the presence of a positive sample. This approach was eventually tested with 30 positive and 30 negative clinical samples. Good agreements between the read-outs from the device and the standard were observed in all samples.

In the next paper, Li et al., built a flexible Terahertz **(**THz**)** metamaterial biosensor for ultra-sensitive detection of Hepatitis B viral DNA based on the metal-enhanced sandwich assay. THz spectroscopy is preferentially sensitive to the dynamic expressions of intermolecular modes and has proven to have potential to detect nucleic acids. With sandwich complexes comprising gold magnetic nanoparticle–rolling circle amplification–gold nanoparticles and a transparent and ultrathin PET substrate patterned with a planar array of asymmetric double-split rings, a larger magnitude of target DNA detection signal amplification was exhibited as compared to the traditional RCA-THz metamaterial biosensor method, which may be due to the gold-mediated nanoparticles’ high refractive index. The final results confirmed that their THz biosensor achieved a LoD as low as 1.27 × 10^2^ IU/ml of serum HBV DNA at concentrations in a range of 1.27 × 10^2^–1.27 × 10^7^ IU/ml.

At last, this Research Topic collects a paper that proposes a unique concentration device to enrich protein biomarkers for sensitive and rapid LFA detection. In this paper, Chen et al., successfully utilized osmotic pressure to concentrate specimens by up to 100-fold. The concentrated specimens were then collected and flown through commercial LFA rapid testing strips to examine the presence of target proteins. Two common biomarkers, chorionic gonadotropin hormone (hCG) and SARS-Cov-2 Nucleocaspid (N protein) were tested to evaluate the effects of their approach. With the osmotic concentration, the signal intensities of 0.02 μg/ml hCG and 0.04 ng/ml N protein were increased from 1.01 to 6.0 and 1.08 to 1.61, respectively. The authors believed their osmotic processor can achieve similar enrichment as the existing techniques.
